# Impact of family environment on mental disorders and quality of life in children with type 1 diabetes mellitus: a cross-sectional study and intervention policy analysis

**DOI:** 10.3389/fped.2025.1516411

**Published:** 2025-03-03

**Authors:** Jing Liu, Jinhong Li, Lichang Li, Kun Zeng

**Affiliations:** ^1^ Department of Pediatric Internal Medicine (Section Two), Dongguan Eighth People’s Hospital (Dongguan Children’s Hospital), Dongguan, Guangdong, China; ^2^ Department of Science and Education, Dongguan Eighth People’s Hospital (Dongguan Children’s Hospital), Dongguan, Guangdong, China

**Keywords:** type 1 diabetes mellitus, anxiety, depression, family environment, quality of life, correlation

## Abstract

**Background:**

Type 1 diabetes mellitus (T1DM) is common in adolescents and negatively affects their quality of life and mental health. This study examines the impact of family environment on mental disorders and quality of life in adolescents with T1DM and analyzes related intervention policies.

**Methods:**

A retrospective analysis was conducted on 75 adolescents with T1DM admitted between October 2020 and December 2023, with 75 healthy adolescents as a control group. Assessments included SCARED, DSRSC, FES, SCL-90, and PedsQL 4.0. Correlation analysis explored the relationships between family environment, anxiety, depression, quality of life and glycosylated haemoglobin (HbA1C).

**Results:**

Significant differences (*P* < 0.05) were found between the T1DM and control groups in family conflict, independence, harmony, and emotional expression. The T1DM group had higher anxiety, depression, and poorer quality of life. Family cohesion was negatively correlated with mental state, anxiety, depression, and HbA1C, while emotional expression was positively correlated with role functioning.

**Conclusion:**

The family environment significantly impacts the mental health and quality of life of adolescents with T1DM. Enhancing emotional expression and family cohesion can improve outcomes, highlighting the need for targeted interventions.

## Introduction

Teenagers with diabetes most frequently have T1DM. It is characterized by a lack of insulin and hyperglycemia that follows, which is brought on by CD4+ and CD8+ T cells’ autoimmune destruction of β-cells and the infiltration of macrophages into the islets (1). In today's rapidly developing society, living standards have been continuously improving, leading to significant changes in dietary habits and lifestyle. Consequently, health problems are increasing. In recent years, the incidence of diabetes has been rising globally, with an increasingly younger demographic. Research indicates that the global incidence of T1DM has been steadily increasing by 2%–3% annually over the past few decades (2). According to statistics from the International Diabetes Federation (IDF), as of 2021, more than 1.2 million children and adolescents worldwide are affected by T1DM, with half of these patients being under the age of 15 (3). In addition, the number of children with T1DM increased to varying degrees in all regions (4). In Europe, Finland and Sweden have the highest incidence, with annual increases of about 2%–5% (5). In the Middle East, children in the Asr region of Saudi Arabia have high HbA1c levels, indicating problems with poor control (6). In Asia, the incidence of T1DM is increasing, especially in rapidly urbanizing countries such as China (7) and India. This presents a substantial burden on both society and families.

Research indicates that the prevalence of mental disorders such as depression and anxiety among adolescents worldwide is on the rise (8), a trend exacerbated among adolescents with T1DM (9). A study from China found that increased hazard ratios of schizophrenia (12.28), bipolar disorder (13.80), major depressive disorder (10.41), ASD (14.52), and ADHD (8.19) in patients with T1DM (10). Brigitta et al. found that the subjective well-being and emotional level of children with T1DM were significantly higher than that of the control group, but the level of physical symptoms and depression was significantly lower than that of the control group (11).

The mental health of T1DM children and adolescents is mainly affected by internal factors, interpersonal relationship and environmental factors (12, 13). Emotional support and effective communication from parents can significantly improve mental resilience and quality of life, while excessive control or lack of support can worsen anxiety and depression (14). Understanding and support from peers, educators and medical staff can also help relieve psychological stress. In addition, adequate medical resources and psycho-educational interventions are critical to mental health.

Among them, the family environment plays a decisive role. The chronic nature of T1DM and its long-term management demands not only exacerbate patients’ economic burden but also intensify their psychological burden. The interaction between chronic diseases and mental health underscores the necessity and significance of exploring influencing factors. Family environment plays a crucial role in the mental health of adolescents with T1DM; factors such as dysfunctional family dynamics, conflicts within the family, parental psychological states, and approaches to disease management are closely associated with anxiety and depression in affected children (15–17). A supportive family environment characterized by close bonds and effective functionality plays a pivotal role in alleviating psychological burdens among young patients. Moreover, the family environment significantly impacts the quality of life (18, 19) and glycosylated haemoglobin (HbA1C) (20) of adolescents with T1DM.

Our research focuses on the influence of specific factors, such as cohesion, conflict and etc., of family environment on mental health, quality of life and blood glucose control of adolescents with T1DM. At present, there are relatively few researches on the influence of family environment factors on the psychology and quality of life of T1DM adolescents, especially the correlation studies between them. This study not only fills this gap, but also makes corresponding analysis for the formulation of targeted intervention policies. We assume that the improvement of adolescent family environment will help reduce the risk of mental disorders and improve the quality of life. This study provides a new therapeutic perspective for clinicians, and provides certain intervention guidance for parents of children with T1DM, helping adolescents to improve their psychological state and overall quality of life in the process of coping with T1DM.

## Materials and methods

### Research sample

We conducted a retrospective analysis based on the collected data, dividing 150 adolescents into two groups: an observation group of 75 patients with Type 1 Diabetes Mellitus (T1DM) and a control group of 75 healthy adolescents who came to our hospital for physical examinations from October 2020 to December 2023. The clinical data collected included gender, age, weight, educational level, family history of diabetes, parents’ educational level, parental divorce, and annual household income. There were 43 males and 32 females in the observation group, aged 7–16 years, and 35 males and 40 females in the control group, aged 8–16 years. We also collected data on the participants’ HbA1c. Table 1 provides comprehensive demographic data. G*power 3.1.9.6 was used to determine the target sample size for inter-group comparison as 128, with 64 cases in each group. The significance level (α) was set at 0.05, β=0.20, and the power (1-β) = 0.80. Results For correlation analysis, the target sample size was 84 cases, and the significance level (α) was set at 0.05, correlation ρH1 = 0.30, and the power (1-β) = 0.80. Due to time and ethical constraints, a total of 150 cases were collected, with 75 cases in each group. The Institutional Review Board gave its approval to this study, which followed the guidelines set forth in the Helsinki Declaration.

**Table 1:** General demographic data of the two groups of adolescents [$\bar{x}\pm s$, M (Q_min_,Q_max_), *n* (%)].

Items		Control group	Observation group	$x^{2}$ */t/Z*	*P*	Cramér's V/r/Cohen’d
Gender	Male	35 (46.67%)	43 (57.33%)	1.709	0.191	0.106
	Female	40 (53.33%)	32 (42.67%)			
Age(years)		10 (7,16)	11 (8,16)	−1.250	0.211	−1.250
Weight/kg		28.56 ± 3.94	28.33 ± 4.23	0.340	0.735	0.056
Education level	Primary school or below	63 (84.0%)	56 (74.67%)	1.992	0.158	0.106
	Middle school	12 (16.0%)	19 (25.33%)			
Family history of diabetes	Yes	4 (5.33%)	10 (13.33%)	2.836	0.092	0.137
	No	71 (94.67%)	65 (86.67%)			
Parents’ education level	Primary school or below	3 (4.00%)	5 (6.67%)	3.196	0.202	0.103
	Middle school	42 (56.00%)	50 (66.67%)			
	University or above	30 (40.00%)	20 (26.66%)			
Parental divorce	Yes	7 (9.33%)	5 (6.67%)	0.362	0.547	0.049
	No	68 (90.67%)	70 (93.33%)			
Annual household income (10,000 CNY)	<5	4 (5.33%)	3 (4.0%)	1.456	0.483	0.070
	5–10	46 (61.33%)	53 (70.67%)			
	>10	25 (33.33%)	19 (25.33%)			
HbA1c(%)		5.08 (4.15,5.61)	7.46 (6.52,8.48)	−10.572	<0.001	−0.863

HbA1c, glycosylated haemoglobin.

Inclusion criteria: Observation group: (1)Diagnosis according to the “Chinese Expert Consensus on Standardized Diagnosis and Treatment of Childhood T1DM (2020 Edition)” (21), with symptoms including polydipsia, polyuria, polyphagia, significant weight loss, and confirmed by laboratory tests, with random blood glucose >11.1 mmol/L; (2) No severe visual, auditory, or language impairments,; (3) Absence of other metabolic disorders; (4) Clinical data integrity. Control group: (1) Absence of other metabolic disorders; (2) No severe visual, auditory, or language impairments; (3) Clinical data integrity. All samples were aged 7–16 years.

Exclusion criteria: (1) Congenital heart disease; (2) Coexistence of other chronic diseases; (3) Significant organ diseases affecting heart, liver, kidneys, etc.; (4) Primary caregivers with behavioral, cognitive, or psychiatric disorders; (5) Coexistence of malignant tumors; (6) Individuals with severe visual, auditory, or language impairments.

## Sampling

### Instruments

This study employed a retrospective cross-sectional research design. Initially, individuals from both groups were assessed using scales to evaluate family environment, mental status, depression, anxiety, and quality of life. The scales measuring family environment, mental status, anxiety, depression, and quality of life were completed by all participants in the observation and control groups. Under the guidance of professionals, all adolescents correctly completed each scale, and data collection was immediately conducted, achieving a questionnaire collection rate of 100%.

The scales utilized in this study are as follows:

Family Environment Scale (FES) (22) consists of 10 subscales, each evaluating a distinct characteristic of the family environment. The subscales are conflict, organization, independence, recreational orientation, control, cohesion, emotional expression, moral-religious emphasis, intellectual-cultural orientation, and achievement. The scale includes 90 items, each rated on a 1 to 5 scale: 1 (not at all), 2 (occasionally), 3 (sometimes), 4 (often), and 5 (always). Higher scores indicate better family relationships. The scale is completed independently by the parents. In terms of reliability, as measured by internal consistency, the originally reported alpha coefficients’ for each subscale ranged from 0.64 to 0.79.

The Symptom Checklist-90 (SCL-90) (23) is a self-reported symptom assessment used to evaluate a wide range of psychological issues and symptoms in adults. Nine core symptom dimensions are measured by its ninety items: phobic anxiety, paranoid ideation, obsessive-compulsive, interpersonal sensitivity, depression, anxiety, anger, and somatic panic. Respondents rate the frequency and severity of their symptoms over the course of the previous week on a 5-point rating system, where 0 means “not at all” and 4 means “extremely.” Greater severity of symptoms across all evaluated aspects is indicated by higher scores. Cronbach's alpha for the SCL-90 has been reported to be 0.77 to 0.90.

The Screen for Child Anxiety Related Emotional Disorders (SCARED) (24) consists of 41 items and is designed to assess anxiety symptoms in children and adolescents. It includes five factors: somatization, panic disorder, social phobia, school phobia, separation anxiety, and generalized anxiety. Every item has three possible scores: 0 for nothing, 1 for occasionally true, and 2 for frequently true. Greater anxiety symptom severity is indicated by higher SCARED scores. Cronbach's alpha ranges from 0.75 to 0.92 for the total scale.

The Self-Rating Scale for Depressive Disorder in Childhood (DSRSC) (25) contains 18 items scored on a three-point scale from 0 to 2. For items 3, 5, 6, 10, 14, 15, 17, and 18, a score of 0 indicates “not at all,” 1 indicates “sometimes,” and 2 indicates “often.” For the remaining items, the scoring is reversed, with 0 indicating “often,” 1 indicating “sometimes,” and 2 indicating “not at all.” This scale employs negative scoring, with higher scores indicating the presence of depressive symptoms. Cronbach's alpha typically ranges from 0.80 to 0.90.

The Pediatric Quality of Life Inventory 4.0 (PedsQL 4.0) (26) comprises four dimensions: school functioning, physical functioning, social functioning, and emotional functioning.The five items that make up school functioning are “I misplace things.” and “I find it difficult to stay on top of my academics.” Physical functioning includes 8 items, such as “I find it difficult to exercise or participate in sports.” Social functioning includes five items, such as “Other kids do not want to be my friends.” Emotional functioning includes five items, such as “I feel sad or depressed.” In total, there are 23 items, each scored on a scale from 0 to 4, representing the frequency of each item occurring in the past month. Better lives are indicated by higher scores. Cronbach's alpha for the overall scale typically ranges from 0.80 to 0.90.

### Statistical analysis

Analysis was performed using SPSS 20.0 (IBM Corp., Armonk, NY, USA). Normally distributed continuous data are presented as the mean ± standard deviation. A student's t-test was used for comparisons between two samples. Non-normally distributed continuous data are expressed as median (Q_min_, Q_max_), and the Mann–Whitney *U* test was used for comparisons between groups. Categorical data are presented as [*n* (%)], and comparisons between two samples were conducted using the chi-square test. Correlation analysis was performed using Spearman's correlation coefficient. A significance level of *P* < 0.05 was considered statistically significant.

## Results

### General data analysis

Between the two groups of adolescents, there were no statistically significant variations in the general demographic data (*P* > 0.05) except the HbA1c value(*P* < 0.05). Specifically, gender distribution, age, weight, education level, family history of diabetes, parental education level, parental divorce status, and annual household income were comparable between the control and observation groups. In the control group, 46.67% were male, with a median age of 10 years, while in the observation group, 57.33% were male, with a median age of 11 years. No significant differences were found in the family history of diabetes, parental education, or income between the two groups. The detailed information is shown in Table 1.

### Comparison of family environment survey scores between the two groups of adolescents

Observation group of teenagers in independence, contradictoriness dimension scores higher than that of control group, the cohesion, the emotional expression dimension score lower than the control group (*P* < 0.05). The detailed information is shown in Table 2.

**Table 2:** Comparison of family environment survey scores between the two groups of adolescents [M (Q_min_,Q_max_)].

Group	FES				
	Cohesion	Emotional expression	Conflict	Independence	Achievement
Control group	8 (4,9)	6 (3,9)	4 (0,5)	5 (2,6)	5 (3,7)
Observation group	6 (3,9)	5 (3,9)	5 (2,7)	5 (2,7)	5 (4,7)
*Z*	−7.203	−2.069	−4.069	−2.316	−0.553
*P*	0.000	0.039	0.000	0.021	0.580
*r*	−0.587	−0.169	−0.332	−0.189	−0.045
Group	FES				
	Intellectual-cultural orientation	Recreational orientation	Moral-religious emphasis	Organization	Control
Control group	3 (2,5)	5 (3,7)	5 (4,6)	5 (4,7)	4 (2,5)
Observation group	3 (2,5)	4 (3,6)	5 (4,7)	6 (5,7)	4 (2,5)
*Z*	−1.776	−1.681	−1.041	−1.749	−0.035
*P*	0.076	0.093	0.298	0.080	0.978
*r*	−0.145	−0.137	−0.085	−0.143	−0.003

FES, family environment scale.

### Comparison of mental state, anxiety, and depression scores between control and observation groups of adolescents

Adolescents in the observation group exhibited significantly higher scores in mental state, anxiety, and depression assessments compared to those in the control group (*P* < 0.05). The detailed information is shown in Table 3.

**Table 3:** Comparison of mental state, anxiety, and depression scores between control and observation groups of adolescents [M (Q_min_,Q_max_)].

Group	SCL-90	SCARED	DSRSC
Control group	118 (89,180)	19 (5,26)	13 (6,18)
Observation group	167 (90,356)	25 (18,32)	14 (8,20)
*Z*	−6.137	−7.664	−3.320
*P*	0.000	0.000	0.001
*r*	−0.501	−0.626	−0.271

SCL-90, The symptom checklist-90; SCARED, The screen for child anxiety related emotional disorders; DSRSC, the self-rating scale for depressive disorder in childhood.

### Comparison of quality of life scores between control and observation groups of adolescents

Adolescents in the observation group displayed significantly lower total scores in overall quality of life compared to those in the control group (*P* < 0.05). The detailed information is shown in Table 4.

**Table 4:** Comparison of quality of life scores between control and observation groups of adolescents.

Group	PedsQL 4.0[$\bar{x}$±s]			
	Physical functioning	Emotional functioning	School functioning	Social functioning
Control Group	68.72 ± 7.12	75.92 ± 6.81	66.12 ± 5.80	56.48 ± 7.74
Observation Group	66.72 ± 7.57	70.15 ± 6.37	57.95 ± 6.82	55.24 ± 7.38
*t*	1.666	5.361	7.905	1.004
*P*	0.098	0.000	0.000	0.317
*Cohen’d*	0.27	0.87	1.42	0.16

PedsQL 4.0, the pediatric quality of life inventory 4.0.

### Relationship between family environment and mental state, anxiety, depression in the observation group of adolescents

The results of the correlation analysis indicate that in the family environment of T1DM adolescents, cohesion is negatively correlated with mental state (r = −0.585), anxiety (r = −0.360), and depression (r = −0.415) at a statistically significant level (*P* < 0.05). Emotional expression is also negatively correlated with depression (r = −0.303, *P* < 0.05), while control shows a positive correlation with depression (r = 0.270, *P* < 0.05). The detailed information is shown in Table 5.

**Table 5:** Relationship between family environment and adolescent mental state and anxiety, depression in the observation group.

Scale	FES									
	Cohesion	Emotional expression	Conflict	Independence	Achievement	Intellectual-cultural orientation	Recreational orientation	Moral-religious emphasis	Organization	Control
SCL-90	−0.585**	−0.137	0.123	−0.097	0.154	0.044	0.101	0.060	−0.067	0.048
SCARED	−0.360**	−0.105	0.002	0.134	0.183	0.102	0.038	−0.104	0.027	0.222
DSRSC	−0.415**	−0.303**	−0.060	0.072	0.188	−0.118	0.079	0.002	0.188	0.270*

The Spearman correlation analysis was used to examine the outcomes. The significance levels are shown by *P < 0.05 and **P < 0.01.

### Relationship between family environment and quality of life in the observation group of adolescents

Correlation analysis results show that within the observation group, family environment cohesion is positively correlated with psychological functioning in quality of life (r = 0.681, *P* < 0.05). Additionally, emotional expression is positively correlated with school functioning (r = 0.699, *P* < 0.05). The detailed information is shown in Table 6.

**Table 6:** Relationship between family environment and quality of life in the observation group of adolescents.

Scale	FES									
	Cohesion	Emotional expression	Conflict	Independence	Achievement	Intellectual-cultural orientation	Recreational orientation	Moral-religious emphasis	Organization	Control
Physical Functioning	0.041	0.166	−0.079	−0.123	−0.082	−0.131	−0.109	0.094	0.018	−0.116
Emotional Functioning	0.681**	0.120	−0.016	−0.068	−0.114	−0.057	0.047	0.009	0.042	−0.167
School Functioning	0.073	0.699**	0.009	0.092	−0.147	0.146	0.025	0.059	0.009	−0.028
Social Functioning	0.040	0.089	0.207	0.043	−0.154	0.067	−0.078	0.090	−0.161	−0.053

The results were analyzed using Spearman correlation analysis.

* *P* < 0.05 and **P < 0.01 indicate significance levels.

### Relationship Between Family Environment and HbA1c in the Observation Group of Adolescents

Correlation analysis results show that within the observation group, family environment cohesion is positively correlated with HbA1c (r = −0.403, *P* < 0.01). Additionally, other indicators have no correlation with HbA1c (*P* > 0.05). The detailed information is shown in Table 7.

**Table 7:** Relationship between family environment and HbA1c in the observation group of adolescents.

Scale	FES									
	Cohesion	Emotional expression	Conflict	Independence	Achievement	Intellectual-cultural orientation	Recreational orientation	Moral-religious emphasis	Organization	Control
HbA1c	−0.403**	−0.226	0.060	−0.010	−0.150	−0.054	0.103	0.153	0.013	0.142

The Spearman correlation analysis was used to examine the outcomes. The significance levels are shown by *P < 0.05 and **P < 0.01.

## Discussion

T1DM is a chronic disease commonly occurring in children and adolescents, often imposing significant economic and psychological burdens on patients and their families. Studies show that emotional disorders including sadness and anxiety are more common in T1DM patients. These disorders have a detrimental impact on the way the disease is managed in children with T1DM (9, 10). Studies by Garey CJ (27) have shown that T1DM is a risk factor for emotional disorders in children and adolescents, with puberty being a high-risk period for the onset of these disorders.

The family environment, being a crucial support system for adolescents with T1DM, has multifaceted impacts on these individuals. Family members not only participate in the disease management of adolescents with T1DM, but the psychological atmosphere within the family can also directly or indirectly affect the patient's mental health. Good family support plays a positive role in both disease management and psychological health of the affected children (28–30).

Our study found that adolescents with T1DM had lower scores in family cohesion and emotional expression, but higher scores in independence and control, compared to healthy adolescents. This suggests that the presence of the disease may lead to abnormalities in the family environment. It could be due to the emotional stress and anxiety faced by adolescents with T1DM and their families. Continuous disease management and uncertainty may impact emotional communication and cohesion among family members. Parents’ heightened concern for their child's health may lead to overprotection or intervention, increasing the level of control within the family environment.

This study also found that the scores for mental health status, anxiety, and depression in the observation group were significantly higher compared to the control group, which is consistent with findings from other studies (9, 31). The reasons for this may include the discomfort caused by blood glucose fluctuations, which can directly affect the emotions and behaviors of the patients, thereby increasing the risk of emotional disorders (32, 33). Long-term disease management imposes significant psychological stress on patients. Relevant research indicates that adolescents with T1DM often experience needle phobia and fear of injection pain (34, 35). Furthermore, due to the demands of disease management, these patients may be unable to participate in social activities like their healthy peers (36), and they may even face discrimination, which can increase feelings of loneliness and potentially lead to social disorders.

Adolescents with T1DM may experience mental health issues as a result of their familial situation (37, 38). Thus, the purpose of this study was to investigate the connection between the mental health, anxiety, and depressive problems in the observation group and the family environment. The findings show that, in teenagers with type 1 diabetes, family control and independence are favorably connected with anxiety and depression, but family cohesiveness and emotional expression are adversely correlated with these diseases. Stated differently, teenagers are less likely to acquire anxiety and depression disorders in families where interactions are closer and more expressive of emotions. Conversely, stronger familial control correlates with a higher likelihood of these disorders.

The reason lies in the poor family environment, where parental anxiety about the disease can increase the risk of emotional disorders in patients. The family environment is a crucial part of the lives of children with T1DM. Due to the unique nature of the disease, some parents may exercise increased control over their children, often neglecting the children's own thoughts and feelings. This can lead to increased family conflict and decreased cohesion, thereby negatively affecting the children's emotions and increasing their risk of emotional disorders (39).

Additionally, the quality of life scores of the T1DM teenagers in the observation group were shown to be considerably poorer than those of the control group's healthy adolescents. This finding is consistent with the results of studies by Lizama and Coolen (40, 41). Adolescents with type 1 diabetes have a positive correlation between their familial environment and quality of life, which is consistent with comparable research findings (42, 43). The reason for this is that adolescents with T1DM are in their developmental stage, with psychological functions that are not fully mature and are easily influenced by their environment. The family environment is one of the primary settings where adolescents spend most of their time. High cohesion families typically provide greater emotional support and understanding, which positively impacts the psychological health of the patients.

Furthermore, studies have shown that children from high cohesion families score lower on psychological scales measuring anxiety and depression (15–17). Open emotional expression among family members facilitates communication and reduces internal family stress, enabling adolescents to better manage their role demands and enhance their role functions. This ability to effectively handle various issues arising from family, academic, and social domains contributes to improved overall well-being (44, 45).

Our correlation analysis showed a significant negative correlation between family cohesion and HbA1c level in adolescents with type 1 diabetes mellitus (T1DM) (r = −0.403, *P* < 0.01). This suggests that stronger family cohesion is associated with better blood sugar control. A supportive home environment may enhance communication and self-management behaviors that lower HbA1c levels. Studies have shown that higher diabetes-specific family conflict (often measured by conflict with parents) is associated with higher blood sugar levels, higher HBA1c (away from goals), lower self-management behaviors, higher levels of depression and anxiety, and lower quality of life. This result is transversal (46, 47) and longitudinal study (37, 48)^.^ Both were verified in Hilliard et al., 2011. For adolescents with type 1 diabetes, parental acceptance was associated with higher adherence and lower HBA1c (49). In contrast to previous studies, our study did not find an association between family conflict and blood sugar control. Our findings highlight the importance of family support in managing T1DM, suggesting that strengthening family cohesion may be key to effectively managing juvenile diabetes. Further research should examine the impact of other family dynamics on health outcomes in this population.

Based on the current research, it is imperative to explore the intervention policies of the family environment for adolescents with T1DM. The following intervention policies are proposed for reference: Firstly, family health education should be provided to improve family members’ understanding of the disease, improve the overall medical literacy of the family, help children develop good living habits, and strengthen nutrition education (50, 51), so as to reduce parents’ control over children and reduce conflicts due to disease control. Second, provide psychological counseling and education: remove the fear, treat disease with a healthy mentality, and at the same time, improve the family cohesion, strengthen the emotional expression of the family members, and help reduce the risk of children suffering from mental disorders. Related studies have found that the Internet can impact children with self-management and promote family communication, which has a good supporting effect (52, 53). In addition, regular family activities, including learning and leisure activities, should be organized to help children manage their disease more effectively and improve their role functions in family, school, and society, so as to improve their quality of life. Finally, establish a complete test evaluation and feedback mechanism. Regular use of standardized tools, such as PedsQL4.0, FES, and SCARED child anxiety-related emotional disorders screening, to evaluate children, sum up the experience and results of the analysis, and provide timely feedback to households is suggested to improve and strengthen the intervention plan.

In recent years, researchers have proposed a variety of intervention strategies for the mental health and quality of life of children with T1DM. These strategies include psychological counseling, family therapy, education, and support groups, aiming to improve the family environment and enhance family support so as to improve the mental health of children. The effects of the different intervention strategies, however, need further study and validation.

While our study provides valuable insights into the relationship between family environment, mental health, and quality of life in adolescents with type 1 diabetes (T1DM), some limitations should be noted. Due to time, ethical and other constraints, we were unable to collect data of the target sample size, which led to the low effect size of our results. The small sample size may limit the generality of our findings, and the fact that the study was conducted in one particular hospital may not reflect the broader adolescent T1DM population. In addition, we did not consider cultural and social factors that may affect family dynamics and mental health. As an observational study, it lacked experimental interventions to establish causality, and retrospective data collection could create potential biases. Finally, relying on self-reported measures may introduce subjective bias, suggesting a need for multiple assessment methods in future studies. Acknowledging these limitations helps put our findings into context and points to areas for further investigation.

## Conclusions

In conclusion, adolescents with Type 1 Diabetes Mellitus (T1DM) are significantly influenced by their familial environment, with factors such as family cohesion and emotional expression positively affecting mental health and quality of life, while family control has an adverse impact. For adolescents experiencing mental health issues and a low quality of life, implementing family-centered interventions is crucial. These interventions should focus on enhancing family cohesion, fostering emotional expression, and reducing family control. Healthcare providers can play a vital role by receiving training in family-centered care, improving communication skills, and incorporating psychological support and tailored education into routine care. By addressing these aspects, healthcare providers can effectively reduce mental health risks and improve the overall well-being of adolescents with T1DM.

## Data Availability

The raw data supporting the conclusions of this article will be made available by the authors, without undue reservation.
